# Exploring key-stakeholder perceptions on non-communicable disease care during the COVID-19 pandemic in Kenya

**DOI:** 10.11604/pamj.2023.44.153.38616

**Published:** 2023-03-30

**Authors:** Sugitha Sureshkumar, Kibachio Joseph Mwangi, Gladwell Gathecha, Kailing Marcus, Bogomil Kohlbrenner, David Issom, Mohamed Rida Benissa, Sigiriya Aebischer-Perone, Nirit Braha, Egidio Candela, Kumar Gaurav Chhabra, Bava Ramachandran Desikachari, Arianna Dondi, Marina Etchebehere, Andre Pascal Kengne, Eduardo Missoni, Feisul Mustapha, Benjamin Palafox, Sanghamitra Pati, Priyanka Paul Madhu, Nasheeta Peer, Jennifer Quint, Reza Tabrizi, Haironi Yusoff, Michel Oris, David Henry Beran, Dina Balabanova, Jean-François Etter

**Affiliations:** 1 Institute of Global Health, Faculty of Medicine, University of Geneva, Geneva, Switzerland,; 2 Department of Non-Communicable Diseases, Ministry of Health, Nairobi, Kenya,; 3 University Hospitals of Geneva, Geneva, Switzerland,; 4 Royal Free Hospitals, National Health Service, London, United Kingdom,; 5 RCCS Azienda Ospedaliero-Universitaria di Bologna, Bologna, Italy; 6 Department of Public Health Dentistry, Nims Dental College and Hospital, Nims University, Rajasthan, India,; 7 The Medical Park (HSMP), Chennai, India,; 8 Faculdade Israelita de Ciencias da Saude Albert Einstein, Sao Paulo, Brazil,; 9 Non-communicable Diseases Research Unit, South African Medical Research Council, Cape Town, South Africa,; 10 Center for Research on Health and Social Care Management - CERGAS, SDA Bocconi Management School, Milan, Italy,; 11 Disease Control Division, Ministry of Health, Putrajaya, Malaysia,; 12 London School of Hygiene and Tropical Medicine, London, United Kingdom,; 13 ICMR-Regional Medical Research Centre, Odisha, India,; 14 Department of Public Health Dentistry, Sharad Pawar Dental College and Hospital, Wardha, India,; 15 Imperial College London, London, United Kingdom,; 16 Non-Communicable Diseases Research Center, Fasa University of Medical Sciences, Fasa, Iran,; 17 Department of Public Health, Faculty of Medicine and Health Sciences, Universiti Malaysia Sarawak, Sarawak, Malaysia,; 18 Division of Tropical and Humanitarian Medicine, Faculty of Medicine, University of Geneva, Geneva University Hospitals, Geneva, Switzerland

**Keywords:** Non-communicable diseases, COVID-19, SARS-CoV-2, health care delivery, health care quality, health personnel, epidemics, pandemics

## Abstract

**Introduction:**

over one third of total Disability-Adjusted-Life-Years lost in Kenya are due to non-communicable diseases (NCD). In response, the Government declared significant commitment towards improving NCD care. The COVID-19 pandemic increased the burden on the already overstretched health systems in Kenya. The aims of this study are to assess whether health care providers perceived NCD care to be optimal during the pandemic and explore how to improve responses to future emergencies.

**Methods:**

this cross-sectional online survey included healthcare personnel with non-clinical roles (public health workers and policy-makers) and those delivering health care (doctors and nurses). Respondents were recruited between May and September 2021 by random sampling, completed by snowball sampling.

**Results:**

among 236 participants (42% in clinical, 58% in non-clinical roles) there was an overall consensus between respondents on NCD care being disrupted and compromised during the pandemic in Kenya. Detracted supplies, funding, and technical resources affected the continuity of NCDs’ response, despite government efforts. Respondents agreed that the enhanced personnel capacity and competencies to manage COVID-19 patients were positive, but noted a lack of guidance for redirecting care for chronic diseases, and advocated for digital innovation as a solution.

**Conclusion:**

this paper explores the perceptions of key stakeholders involved in the management of NCDs in Kenya to improve planning for future emergency responses. Gaps were identified in health system response and preparedness capacity during the pandemic including the perceived need to strengthen NCD services, with solutions offered to guide resilience efforts to protect the health system from disruption.

## Introduction

The COVID-19 pandemic reduced the capacity of health systems to address non-communicable diseases (NCD) and increased the burden on already overstretched health systems in many settings [1]. People with NCDs (cardiovascular disease, diabetes, immunological disorders, oncological pathologies, etc.) were at risk of severe COVID-19 infection and less favourable outcomes from COVID-19 [2,3]. On account of this vulnerability, people with NCDs may have avoided seeking or following through with health care, or had reduced access to health facilities, adversely impacting their ability to manage their condition [4,5].

Populations in low- and middle-income countries (LMIC) were particularly affected by this disruption of services [6]. Many LMICs have not reported on the status of NCD care during the pandemic, and the impacts on NCD care have been marginally explored in these settings [6,7]. Nevertheless, a report on 39 participating Member States of the African Union surveyed during the pandemic cited serious concerns about health systems' capabilities to provide effective NCD care. This source reported the challenges to the implementation and monitoring of NCDs, Injuries and Mental Health policies, pertained to limited resources and capacity [7].

Between 1990 and 2017, Disability-Adjusted-Life-Years (DALYs) due to NCDs rose to 67% in sub-Saharan Africa, the region that faces the greatest epidemiological and economical challenge from NCDs amongst LMICs [8,9]; e.g. over a third (37%) of total DALYs lost in Kenya are due to NCDs [10]. Cardiovascular diseases are the leading cause of NCD mortality in this country and their prevalence increased sharply in the last decade [11]. In response, the Government of Kenya has declared significant commitment towards improving NCD services [10]. Nevertheless, this remains a challenge in view of the high burden of communicable diseases (HIV/AIDS, lower respiratory tract infections, and diarrhoeal disease) [12]. With a double burden of disease (i.e. communicable and non-communicable diseases), the health system which has limited resources tends to be overstretched.

With over 300,000 confirmed cases of COVID-19 and 5678 deaths, deaths as of October 2022, [13] Kenya maintained relatively low transmission rates, as did other sub-Saharan African nations, in comparison with Northern Africa and South Africa [14]. The Kenyan health care system did, however, experience access issues pertaining to electronic records, medicines dispensing, and decreased patient confidence in hospital safety [15].

Studies from other countries, looking into the perceptions of patients living with-NCDs, concluded they felt they received inadequate NCD care during the pandemic [16,17]. However, the health care provider perspectives of the status of NCD care has not been explored. Given the government's commitment to NCD care, as well as the relatively lower burden of COVID-19 in Kenya, the aim of this study was therefore 1) to assess whether or not health care providers thought that NCD care was maintained to expected standards during the pandemic, 2) to ascertain changes in practices, if any, to counter future threats to NCD care during times of crisis, and 3) to ascertain changes in practices to counter future threats to NCD care during times of crisis. Perceptions among professionals in clinical and non-clinical roles were examined to compare any differences attributable to their differing experiences and tasks.

## Methods

For the purpose of this research, we formed an international study group that worked in collaboration with the Division of Non-Communicable Diseases in Kenya's Ministry of Health (MoH). The larger study group and study were, however, independent from the MoH.

**Participants and sampling:** participants in this cross-sectional, quantitative study included health personnel with non-clinical roles (public health workers and policy-makers, hereafter referred to as ‘non-clinical workers’, n=138), and those delivering health care (doctors and nurses, hereafter ‘clinical workers’, n=98) (Table 1). The inclusion criteria were as follows: employed in an official public health capacity or in a health policy/governing capacity, or in a clinical capacity in Kenya; or employed by a Non-Governmental Organization in a public health or clinical capacity; age >18 years old, and provided informed consent.

**Table 1:** description of sample

Total Respondents n=236				
Regions	Population in Province (% of total 47,564,296)	Respondents in each Province (% of total 236)	Proportion in a Clinical setting %	Proportion in a Non-clinical setting %
Central	5,482,239 (12%)	19 (7%)	50	50
Coast	4,329,474 (9%)	18 (7%)	39	61
Eastern	6,821,049 (14%)	29 (12%)	45	55
Nairobi	4,397,073 (9%)	52 (22%)	35	65
North Eastern	2,490,073 (5%)	2 (1%)	100	0
Nyanza	6,269,579 (13%)	24 (10%)	33	77
Rift Valley	12,752,966 (27%)	48 (16%)	66	34
Western	5,021,843 (11%)	14 (6%)	57	43
not disclosed	-	30 (17%)	12	19
**Profession**			**N=**	**%**
Non-clinical worker			138	58
public health worker			75	54
health policy worker			63	46
Clinical Worker			98	42
Doctor			64	65
Nurse			34	35
**Years of work experience**			**N=**	**%**
Average years (SD)			11.7	±8
≤5y			42	24
>5y ≤10y			53	31
>10y ≤15y			39	23
>15y ≤20y			19	11
>20y			19	11

The Kenyan MoH provided a list of randomly selected staff within health policy units, clinical facilities, and public health departments, with e-mail addresses. The list comprised a sample of 247 health professionals chosen by the MoH, doctors and nurses throughout Kenya, NCD coordinators in regional public health offices, and policy makers within the ministry.

We sent out e-mail invitations to complete the online questionnaire to all these 247 people and used the LimeSurvey platform to collect the data between May and September 2021. Participants were asked at the conclusion of the questionnaire whether they would forward the survey link to relevant individuals in their respective professional networks (i.e., snowball sampling). Participants' opinions were from their individual capacity and not necessarily representing their organizations; no information allowing for the identification of participants was requested and informed consent was sought on the online platform.

**Measurements:** we created an *ad hoc* online questionnaire assessing the participants' perceptions, needs and expectations. The questionnaire was developed after a review of the relevant literature and by conducting individual telephone interviews to collect the perceptions and views of 4 Kenyan health care personnel accessed through the Kenyan MoH. A preliminary version of the online questionnaire was then pre-tested with 4 public health experts in 2 rounds of pre-tests. The questionnaire covered the following 5 themes: 1. Health system preparedness (policy readiness and implementation capacity), 2. Emergency preparedness (processes in place to counter adverse public health scenarios to NCD care), 3. Allocative efficiencies (distribution and redistribution of human, economic, and technical resources), 4. Perception on government policies and responses (ideas and opinions of officials within the health governing structure), 5. Innovation (possible future initiatives to address adverse public health scenarios to NCD care).

The questionnaire included 49 multiple-choice questions that covered NCD care and COVID-19 response in Kenya (23 questions), and health system preparedness and adaptability (26 questions) (Annex 1). There were 2 distinct questionnaires, one for non-clinical workers and one for clinical workers. Both questionnaires included a common core of questions, but questions involving patient interactions were excluded from the non-clinical worker questionnaire, and questions surrounding policy making decisions were excluded from the clinical worker questionnaire.

**Data entry and analysis:** data collected through the online questionnaire was imported from LimeSurvey into STATA (release 17.0, StataCorp LLC, College Station, TX). Data were kept within a secure server by the University of Geneva. We used means, standard deviations, percentages, and frequencies to describe sample characteristics, and chi-square tests to compare proportions.

**Ethics:** the study was approved by the Institutional Research and Ethics Committee (IREC) of Moi University, Kenya on 04/02/2021, with all procedures being in accordance with its ethical standards (protocol number MTRH/Mu-IREC FAN 3786). The study was implemented as described in the approved protocol, without deviation.

**Funding information:** the authors received no financial support in addition to their salaries for the research, authorship, and publication of this article. There are no financial or non-financial competing interests (political, personal, religious, ideological, academic, intellectual, commercial or any other) to declare in relation to this manuscript.

## Results

We collected data from 236 participants, either directly from our requests or through snowballing, 42% of the respondents were in clinical roles and 58% were in non-clinical roles (54% of which were public health officials and 46% health policy workers working in central and regional government health offices). Nairobi region was the county with the largest number of respondents (22%), and North Eastern Province the lowest number of respondents. The geographic distribution of the sample roughly corresponded to the distribution of the Kenyan population across provinces (Table 1) [18]. Among the respondents, 61% of the clinical group and 100% of the non-clinical group answered >75% of the questions. The response rate was not possible to calculate due to the nature of the anonymous snowball sample (Table 1).

**NCD status:** a quarter (24%) of clinical workers felt that NCD services in Kenya were either ‘good’ or ‘excellent’, while 41% of non-clinical respondents felt the same (p<0.001). Almost two thirds (63%) of clinical workers were not aware that an integrated HIV/TB/Family Planning and NCD plan was in place in Kenya. Over half (59%) of non-clinical workers considered that the health system gave ‘minimal’ priority toward NCD care prior to the pandemic. Half (52%) of the respondents stated that this priority changed during the pandemic because of the heightened risk of severe disease in patients with NCDs (Table 2).

**Table 2:** summary of survey findings- NCD status in Kenya

Total respondents n=236						
Survey Question		Response	Clinical worker n=98	Non-clinical worker n=138	P-value	Chi2
**NCD Status in Kenya**						
**1**	Rate NCD care in your country	good +excellent	20(24)	40(41)	<0.001	22.91
**2**	Are you aware of an integrated HIV/TB/family planning and NCD plan in Kenya?	no	36(63)	43(46)	0.002	12.36
**3**	Would you agree/disagree that the traditional delineation of Infectious and NCD care should be eliminated to provide the holistic care that seems imperative in countering damaging outcomes during infectious epidemics?	agree	31(100)	23(100)	NA	NA
**4**	Has children's care in your institution been compromised in any way?	yes	14(40)	10(59)	0.118	4.28
**5**	What priority was NCD care accorded in your country prior to the COVID-19 pandemic?	minimal	NA	39(59)	NA	NA
**6**	Did this priority change considering the heightened risk of severe disease among patients with pre-existing conditions?	yes	NA	28(52)	NA	NA
**COVID-19 health system response in Kenya**						
**7**	What was the amount of beds created for the surge response?	not many	20(43)	22(56)	0.007	9.75
**8**	Were patients who otherwise would receive a cubicle/isolated space in a department/ward, now being placed in shared space/ward beds?	yes	33(56)	NA	NA	NA
**9**	Were there COVID-19-dedicated hospitals in your city/region/country?	yes	NA	21(75)	NA	NA
**10**	Were multidisciplinary teams created intentionally for working in COVID-19 wards? (ie; redistribution of specializations to internal medicine, respiratory medicine, ICU for the care of COVID-19 patients)	yes	35(71)	55(81)	<0.001	18.98
**11**	Were health professionals briefed on infection protection control and parallel public health measures?	yes	54(100)	79(100)	NA	NA
**12**	Was the dedicated personnel often rotated so as not to tire them?	yes	37(69)	60(79)	0.178	1.81
**13**	Did the dedicated personnel have any training focusing on COVID-19?	yes	50(100)	67(100)	NA	NA
**14**	Did you have the feeling that there was some confusion about how to treat a COVID-19 patient: e.g. frequent changes in drug indications and dosages, protocols?	yes	45(100)	52(79)	<0.001	10.92
**15**	If there was a redistribution of doctors and nurses unfamiliar with high intensity internal medicine care, were they placed on COVID-19 patient wards regularly (long enough to learn protocols and manage well)?	yes	11(23)	36(42)	0.03	6.96
**16**	Do you worry that NCD patients who have not been followed up adequately due to the crisis (and not clinically afflicted by COVID-19) will have worsened outcomes when it comes to their NCD pathology due to this compromised care?	yes	38(100)	65(100)	NA	NA

Note: frequency(percentage); p-value from chi2; missing p-value and chi2 due to no comparison group or expected value <5

**COVID-19 response:** more non-clinical (56%) than clinical workers (43%, p<0.001) stated that enough beds were created for COVID-19 patients during the pandemic (Table 2). Both groups confirmed unanimously (100%) that infection control and parallel public health measures were widely known to health professionals. According to a majority of participants (76%), multidisciplinary teams were created, and often rotated in shifts so as not to exhaust them (Table 2). Although there was strong agreement (100%) that training focusing on COVID-19 was given and adhered to, only a minority (23% of clinical workers versus 42% of non-clinical workers, p<0.001) stated that rotated clinicians were redistributed to COVID-19 wards long enough to learn protocols and manage the situation well. Many respondents (90%) reported that they observed some confusion regarding how to treat COVID-19 patients.

Regarding the well-being of NCD patients who had not been followed-up adequately due to the pandemic (and who were not clinically afflicted by COVID-19), all respondents (100%) were concerned that this group would have worsened outcomes. All respondents (100%) agreed that the traditional delineation of care for infectious diseases and NCD should be eliminated to provide the holistic care that seems imperative in countering damaging outcomes during infectious disease outbreaks (Table 2).

### Health system preparedness and adaptation during the COVID-19 pandemic

**Health system disruption:** a large share of all respondents (59%) rated the frequency of the disruption to chronic disease management as ‘occasional’ or ‘very often’ (no difference between groups), and 36% rated service disruption as ‘severe’ (Table 2 suite).

**Table 2(suite):** summary of survey findings- Kenyan health system preparedness and adaptation

Total respondents n=236						
Survey question		Response	Clinical worker n=98	Non-clinical worker n=138	P-value	Chi2
Kenyan Health System Preparedness and Adaptation						
**1**	Did you feel the political decision makers in your country were aware of the risk to frontline workers?	not at all	NA	23(29)	NA	NA
**2**	Did your healthcare workers receive adequate personal protection equipment dependant on their workplace setting?	no	47(65)	55(49)	<0.001	15.93
3	If you were on the frontline (in emergency departments, on respiratory, or ICU wards) taking care of Sars-CoV-2 positive patients, did you receive adequate personal protection equipment (PPE)?	a little + quite a bit	50(64)	NA	NA	NA
		completely	25(32)	NA	NA	NA
**4**	How would you rate the severity of NCD service disruption (including elective surgery, follow-up clinics, routine care) during the pandemic?	occasional + very often	44(58)	55(59)	0.526	0.40
		severe	24(32)	37(40)	NA	NA
**5**	Was the provision of individual protection devices adequate or inadequate?	adequate	16(22)	NA	NA	NA
**6**	Were there contingency plans to shield the system in case of an external disruption like COVID-19?	no	59(69)	50(50)	0.003	16.10
**7**	Do/did you anticipate an over burden on the NCD services when routine care eventually resumed?	yes	71(96)	59(59)	<0.001	30.97
**8**	Do/did you have plans for gradual/spaced out resumption of routine care?	yes	31(39)	65(64)	<0.001	27.84
**9**	Do you plan to evaluate the medical impact of this pandemic on NCDs?	yes	NA	49(49)	NA	NA
**10**	Do you plan to evaluate the social and economic burden of this pandemic on NCDs management?	yes	NA	59(62)	NA	NA
**11**	Is there a plan in place to screen the potential mental health impact of the pandemic?	yes	NA	52(58)	NA	NA
**12**	As those with NCDs are the most vulnerable subgroups of patients, do you plan to target increased prevention, health education programmes towards them?	yes	NA	37(66)	NA	NA
**13**	As those with NCDs are the most vulnerable subgroups of patients, do you plan to target increased outreach initiatives (using volunteers, increased personnel, etc.) and address accessibility (using telemedicine, mobile pharmacies, etc.)?	yes	NA	50(73)	NA	NA
**14**	Would you agree/disagree with the need to ensure that whole populations should have increased health promotion, prevention schemes addressed to them to decrease vulnerabilities and predispositions to NCDs, with the goal of optimizing prognoses in the time of infectious epidemics?	agree	37(100)	60(100)	NA	NA
**15**	Would you consider using digital health applications for medical information sharing, especially for patients in multidisciplinary care settings?	yes	34(100)	44(94)	0.133	2.25
**16**	Would you agree that digital health (eHealth or mHealth) can be integrated into your healthcare infrastructure to better care for your most vulnerable patients in crises such as this?	agree + strongly agree	38(69)	47(63)	0.447	0.58
		disagree + strongly disagree	17(31)	28(37)	NA	NA
**17**	To better maintain the normal flow of follow-ups and care of your country's NCD patients, how likely are you to depend on telehealth, digital health, mobile health applications?	very much	36(84)	37(43)	<0.001	21.77
**18**	Taking into account health literacy, access to mobile devices, and financial aspects, would digital health be an option for NCD patients to experience more autonomy over their care?	yes	50(83)	70(82)	0.205	3.16
**19**	Did your country's health system cope with the redistribution of resources (both human and economic) at the cost of continuous care of NCD patients?	somewhat	NA	15(22)	NA	NA
**20**	Did you reorient national/regional guidelines and protocols to concentrate services in a setting suited to high-volume, high-acuity care available 24 hours per day?	yes	27(38)	38(37)	0.945	0.11
**21**	Was redirecting chronic disease management to focus on maintaining supply chains for COVID-19 an option?	yes	38(43)	48(48)	0.804	0.44
**22**	If redirection of chronic care was an option, how successful was it in terms of delivery of care?	met standards	19(29)	55(60)	<0.001	24.92
**23**	If redirection of chronic care was an option, how successful was it in terms of output?	below standards	29(53)	28(44)	0.01	11.03

Note: frequency(percentage); p-value from chi2; missing p-value and chi2 due to no comparison group or expected value <5

A majority of the clinical workers felt that personal protection equipment (PPE) was not adequately distributed (‘a little’ or ‘quite a bit’ = 64%, ‘completely’ = 32%). There was a significant difference between groups about the perceived successfulness of the redirection of care for chronic diseases, with 29% of the clinical staff and 60% of the non-clinical workers responding that the delivery of care “met standards” in this situation (p<0.001). Regarding the outputs from the redirected chronic care, 53% of clinical workers and 44% of non-clinical workers stated that this was below standards (p=0.01). One third of both groups observed that national/regional guidelines and protocols were reoriented to concentrate services on acute care (Table 2 suite).

Two-thirds of clinicians reported that no contingency plans were in place to shield the health system from external disruptions, an opinion shared by half of those in the non-clinical field (p=0.003). Almost all clinical workers (96%) anticipated that NCD services would be overburdened when routine care eventually resumed, and 59% of non-clinical workers believed the same (p<0.001). Regarding their thoughts on the existence of plans for gradual resumption of routine care, 39% of clinical workers and 64% of non-clinical workers answered they believed that such plans existed (p<0.001) (Table 2 suite).

**Health promotion and prevention of NCDs:** both groups agreed on all aspects of planning for health promotion and preventative medicine: 100% of both groups agreed that whole populations should have increased health promotion/prevention schemes to decrease vulnerabilities to NCDs (Table 2 suite). The majority (73%) of non-clinical workers stated that they would strengthen outreach initiatives for patients with NCDs, such as increased community health worker activity and health education programmes. Two-thirds (66%) of non-clinical workers endorsed the need for increased prevention and health education programmes for NCDs (Table 2 suite).

**Digital health:** almost all participants (>94%) saw digital health interventions as optimal for medical information sharing in multidisciplinary care settings (100% of clinical workers, and 94% of non-clinical workers, p=0.1), and thought that these technologies would allow NCD patients to experience more autonomy (82% and 83% of non-clinical workers and clinical workers, p=0.2). Most participants (66%) agreed that eHealth/mobile health (mHealth) could be integrated into the healthcare infrastructure to better care for the most vulnerable patients (no between-group difference). Most clinicians (84%) answered that they were “very much” likely to depend on telehealth, digital health, and mobile health applications to care for NCD patients and maintain the flow of follow-ups (Table 2 suite).

## Discussion

Given the significant gap in knowledge surrounding best practices for the adaptation of NCD care delivery during a public health crisis, this study sought to explore the perceptions of professionals in clinical and non-clinical roles regarding these factors in the context of the COVID-19 pandemic in an African country, Kenya. The existing literature fails to address the intricacies of health service delivery for NCD care and this study adds value by seeking the perceptions of stakeholders in a position to alter decision making and service delivery criteria. The analysis spanned themes related to the delivery of NCD care, the response of the health care system, the adaptation of the NCD services during the pandemic, and practices to counter future deterioration in NCD care in future times of crisis.

This study identifies several major findings. First, healthcare stakeholders in Kenya underscored preparedness issues including NCD service disruption, post-pandemic volume burdens and lack of contingency plans for NCD care, as well as the need for more health promotion and prevention schemes to decrease vulnerabilities and predispositions to chronic illness in the population. Participants positively commented on personnel capacity and competencies to manage COVID-19 patients, though concerns about the redirection of management and care from chronic to acute services and confusing clinical management of COVID-19 was evident. It was apparent that allocative efficiencies, including hospital beds and multidisciplinary teams, although perceived as sufficient, were not properly conveyed to those delivering care. Finally, both clinicians and non-clinicians emphasise the need for digital transformation of healthcare to promote health awareness, education, knowledge management, and information sharing for healthcare professionals and to improve access to care.

***NCD care and health system preparedness:*** clinical and non-clinical respondents were most concerned about the severity of NCD service disruption, post-pandemic volume burdens, and lack of contingency plans for NCD care. This study shows that health workers felt that the Kenyan healthcare system reacted to the crisis, with the creation of multidisciplinary teams, training and equipping of health personnel, rotations to avoid exhaustion, and plans for the resumption of healthcare after the crisis. In particular, participants agreed that the enhanced personnel capacity and competencies to manage COVID-19 patients were adequate. The positive perceptions are surprising given the poor health care system capacity on the African continent, although this could be due to low case load [19]. They, however, noted a lack of guidance for redirecting care for chronic diseases, COVID-19 case management, as well as inadequate hospital bed creation. The confusion relayed about treatment plans can be explained as guidelines and protocols evolved rapidly in response to the steady flow of new scientific knowledge during the pandemic [20].

Most respondents were concerned about worsening of health outcomes due to compromised care with inadequate follow-up, and disruptions of both elective procedures and continuous care during the pandemic in Kenya. These disruptions occurred in a context where resources for healthcare at regional level were already scarce before the pandemic [21], augmenting the problem considering the diminished quality of care evident in even robust and well-resourced health systems [22]. It is understandable that detracted supplies, funding, and technical resources affected the continuity of NCDs response, even though Kenya listed NCD services as essential and published a guideline to optimize NCD care during the COVID-19 pandemic [20].

***Allocation and redistribution of resources:*** respondents commended the wide dissemination of infection prevention control, safe practice, and personnel training, and parallel public health measures. However, they were critical about the reorientation of resources concentrated on high-acuity services, with almost 60% of all respondents rating the disruption to chronic disease management as ‘occasional’ or ‘very often’. Even though additional beds were created for COVID-19 patients, 43% of clinical workers thought this was insufficient. This problem was also seen throughout the African continent, as earlier studies called for better preparedness during crises using, for example, modular services to sustain health service delivery [23,24].

***Health promotion:*** all respondents favoured increased health promotion and prevention to decrease NCDs. This is widely held to be key for optimal health outcomes, as is seen in the roadmap for a strengthened coalition for African Member States from the African Centres for Disease Control and Prevention [7]. The disproportionate mortality and or severe disease in COVID-19 patients who had pre-existing conditions underlines the need for heightened health promotion.

***Holistic care:*** there was a strong agreement between participants on the need for reducing the traditional delineations between the treatment of infectious and NCD management. This is consistent with recommendations that providing holistic care combining NCD and infectious disease care would address the increased susceptibility to communicable disease in individuals with NCDs [25]. This call for integrating NCDs into other primary health platforms for infectious diseases is supported by the multimorbidity nature of the country [10] and should augment the need to provide holistic universal care that stresses a patient-centred focus as opposed to disease based silos.

***Digital health:*** almost all respondents agreed that digital health interventions would be useful for medical information sharing and to manage the follow-up of patients. Research suggests that information technologies may be a way forward in managing both acute and chronic illness [26]. especially in the context of disasters or crisis [27]. Initiatives incorporating digital technologies may be useful in providing integrated health care platforms to accommodate the increasing burden of NCDs [28]. Most participants stated that these technologies would allow NCD patients to experience more autonomy, echoing extant studies that also show how electronic records platforms and eHealth/ mHealth initiatives may improve efficiency in care [28,29]. The questions posed regarding digital health did not explicitly ask for their use in NCD management during the COVID-19 pandemic, yet the context in which the respondents answered probably affected their views regarding this.

Still, telehealth, digital health, and mobile health were not universally accepted by participants, although it has been noted previously that the uptake of these technologies is heterogenous in Kenya [30,31]. In the field, these technologies may reduce disease and prevent overloading of the healthcare system [26], so long as disparities in digital literacy, access, and resources (in particular stable connections) are addressed [32,33].

***Comparison of Clinical and Non-clinical workers:*** the two groups of respondents disagreed about several issues pertaining to the quality of NCD services, redirection of chronic care, and plans for gradual resumption. The continuity of NCD service delivery during the COVID-19 pandemic in Kenya was steered by directives and interim guidelines, as relayed to us by our colleagues in the MoH in Kenya [34]. The implementation of guidelines was disjunct and heterogenous, causing the two groups to have differing opinions and perceptions of policies that were put in place to bolster the system during the pandemic [34]. Participants also diverged in perceptions of the quality of NCD care in Kenya, with fewer clinical than non-clinical workers feeling that NCD services were good. The quality of NCD services in Kenya was surveyed nationally just prior to the pandemic, and results revealed a limited readiness of facilities to manage NCDs [35].

Clinicians in our sample anticipated NCD services would be overburdened when routine care eventually resumed, but this view was less prevalent among non-clinicians. This finding is consistent with reports about clinicians, globally, being concerned that NCD services would suffer in the aftermath of the pandemic [6,25,26]. Fewer clinicians than non-clinicians (respectively a third and over a half) reported that standards were met for the redirection of delivery of chronic care. Previous assessments show that redirection of care is imperative for the continuous care required for chronic patients [22].

Information dissemination is key during crisis and emergency situations. Our findings showed that during the COVID-19 pandemic in Kenya, information about the redistribution of human and material resources was not optimally conveyed to those delivering care, and there was a disjoint awareness between clinicians and non-clinicians of plans to resume NCD care after the crisis. This shows a need for improved information dissemination. Prior studies showed that better knowledge sharing practices can improve awareness, adoption, and use of evidence, and that this in turn can improve policy implementation by adherence to specific communication and/or dissemination strategies [36].

The reasons for this disconnect between the view of clinical and non-clinical respondents on themes relating to quality of NCD care, the impact of COVID-19, and planning redirection are likely related to organizational factors, worker experiences, access to information and networks of colleagues, motivations and tasks, as suggested by other studies [37,38] In the future, we recommend that the two groups forge a constant working collaboration, to ensure that the resultant policies and directives have the intended impact. This is especially important as this study has shown stakeholders' knowledge of health system bottlenecks and emphasizes the necessity of their involvement in emergency preparedness and planning to ensure NCD services are not disrupted in a similar manner in future crises.

**Limitations:** we relied on self-reports, which reflect the subjective knowledge and experience of the respondents and the information they have access to, may not accord with an objective evaluation of the situation. The response rate could not be calculated, as responses were anonymous and the number of requests sent from snowballing from the initial 247 notifications is unknown. Using snowball sampling may have affected the representativeness of our sample and the generalizability of our results. However, though we cannot assert that our sample reflects the population of clinical and public health workers in Kenya, the geographic distribution of our respondents across provinces roughly matched the distribution of the general population. Given the disruptions during the pandemic, and the repurposing of staff to halt and reverse the burden, the list of random participants provided by the MoH completed with subsequent snowball sampling were the most sensible and only feasible techniques were to reach practitioners with relevant information during the pandemic. Our study does not reflect the views of patients and other stakeholders not included in our sample and the lack of a control group (e.g. pre-pandemic) limits our ability to interpret the results.

## Conclusion

This study sought to contribute to strengthening NCD care in Kenya during times of crisis. It provides new and original insights into how NCD care provision and capabilities managed among COVID-19 challenges. Exploring the perceptions of key actors involved in the management of NCDs in Kenya is vital for effective planning of viable responses to crises in the future. The study identified gaps and strengths in the health system response and preparedness capacity during the pandemic. It also highlights the perception that health system development and infrastructure is required in strengthening NCD services country-wide, with necessary continuation of essential health services as a primary pillar of strategic preparedness, readiness and response plans. This may help to inject some resilience, reduce excess mortality and morbidity, reduce the disruption as well as foster quick recovery of these systems during pandemics and other crises.

### What is known about this topic

- *The COVID-19 pandemic provided an unprecedented challenge on the resilience of health care systems globally;*
- *people living with non-communicable disease were at increased risk of hospital admission, severe disease, and death from COVID-19 and their perceptions have cast light on diminished non-communicable disease services and standards during the pandemic;*
- *NCD systems on the African continent were not resilient enough owing to lack of policy attention and financing and thus could possibly not withstand an external shock*.

### What this study adds

- *Assessments and comparisons of health professionals' opinions and perceptions in Kenya on non-communicable disease care during the pandemic;*
- *Highlights the perception that strengthening NCD services requires health system development and infrastructure, and prioritizing continuation of essential health services as a primary pillar of strategic plans;*
- *Stakeholders knowledge of health system bottlenecks and emphasize the necessity of their involvement in emergency preparedness and planning to ensure NCD services are not disrupted in a similar manner in future crises*.

## Annex

**Annex 1**: COVID-19-elephant questionnaire (PDF 677 KB)
